# Remain or return? An empirical study of influencing factors on the return of Chinese international students during the COVID-19 pandemic

**DOI:** 10.3389/fpsyg.2022.1067184

**Published:** 2022-11-23

**Authors:** Keming Zhang, Neng Zeng, Kesen Zhang

**Affiliations:** ^1^School of Educational Science, Nanjing Normal University, Nanjing, China; ^2^School of Teacher Education, Xuzhou University of Technology, Xuzhou, China; ^3^School of Economics and Management, Nantong University, Nantong, China

**Keywords:** COVID-19 pandemic, rational choice theory, international students, influencing factors of returning home, public health crisis

## Abstract

**Background:**

COVID-19 is now a global public health crisis with unprecedented political, economic, and social consequences affecting nations across the world. It also has a profound impact on the mobility of international students. When the COVID-19 was under control in China, and it was spreading dramatically in the United Kingdom, Chinese international students studying in the United Kingdom have been caught in a double bind over whether to return home or not.

**Objective:**

This study aims to explore the factors that influenced Chinese international students’ choices of return during the COVID-19 pandemic when the COVID-19 was under control in China, while it was spreading dramatically in the United Kingdom.

**Methods:**

Taking Chinese international students studying in the United Kingdom as an empirical case, this study used qualitative and quantitative research methods to explore the factors that influenced their choices of return. Based on the Rational Choice Theory and qualitative analysis of text data, this paper constructed the influencing factors model of returning to China. On this basis, we developed a questionnaire and collected data from 1,333 students in late April and early May 2020. Binary Logistic Regression with 95% CI for odds ratio (OR) was used to identify significant factors.

**Results:**

The reserve of epidemic prevention supplies (OR = 0.712), transportation expenses (OR = 0.618), and quarantine expenses (OR = 0.702) negatively affected the return choice of overseas students. The supply of daily necessities (OR = 1.495), the anti-epidemic policy of the United Kingdom (OR = 1.684), and the demand for job hunting after graduation (OR = 1.661) had positive effects.

**Conclusion:**

The institutional rationality had the biggest promoting effect, replaced development rationality, and became the most important factor for overseas students to return to China during the COVID-19 pandemic. Economic rationality, which has a significant negative effect, is the biggest obstacle to returning home. These conclusions have policy implications for governments’ response to the COVID-19 epidemic and improvement of the quality of services for overseas students.

## Introduction

The COVID-19 pandemic has caused millions of deaths since the end of 2019 and is a major global public health threat (Shi et al., 2022). The epidemic not only endangers people’s lives but also impacts the international economic, political, and social order. It also has a profound impact on the pattern of international higher education, especially the mobility of international students (Mok et al., 2021). On the one hand, due to the impact of the epidemic, the willingness and number of students to choose to study abroad have been greatly reduced, and many people have postponed or canceled their plans to study abroad (Marginson, 2020). On the other hand, facing the more complicated international environment, especially the different COVID-19 epidemic prevention and control situations in different countries, overseas students are trapped in a double bind (Ma and Miller, 2021). Whether or not to return to their country has become the most important practical concern. When returning home, they may have to bear biggish economic costs, and their schooling will be adversely affected (Ma and Miller, 2021); if they do not return home, their physical and mental health will be threatened by the pandemic (Firang and Mensah, 2022). Some studies have shown that college students show different degrees of anxiety (Irfan et al., 2021), fear, and depression (Reznik et al., 2022) during the COVID-19 epidemic. Therefore, whether to go or stay is not a simple choice, but is affected by many complex factors.

At present, there are abundant researches on the influencing factors of overseas students’ return home country. Previous studies have discussed from the aspects of personal development, economy, politics, cultural adaptation, and emotional connection (Cheung and Xu, 2015). Research shows that personal development prospects (Mok et al., 2022), such as working conditions, professional prestige, and social status (Mahroum, 2000; Bauder, 2015), are the primary consideration for international students. Economic factors are also important indicators for many overseas students to choose whether to return home (Mohamed and Abdul-Talib, 2020). It includes not only macro factors such as the country’s social and economic development level (Adnett, 2010; Paile and Fatoki, 2014) and national scientific research investment (Guochu and Wenjun, 2002) but also personal economic benefits (Güngör and Tansel, 2008; Dustmann et al., 2011). In terms of politics and policy, the country’s political stability and sound legal system can attract overseas students to return home (Gribble, 2008; Mohamed and Abdul-Talib, 2020). On the contrary, if the domestic political situation is unstable, life safety and property cannot be guaranteed, the international students will not choose to return home. The management policy (Gribble, 2008), incentive policy (Güngör and Tansel, 2008), national compulsory service policy (Soon, 2010), military service policy (Tansel and Güngör, 2003), visa policy (Yu, 2016), and other policies have different forces on the return of international students. The adaptation to foreign culture (Alberts and Hazen, 2005) and the degree of integration into social life (Tharenou and Seet, 2014; Bonifazi and Paparusso, 2019) also affect the choice of overseas students. The social relations between overseas students and their relatives and friends at home and abroad play a key role in their choice of returning home. The social relations be the domestic pull to enhance their willingness to return home (Gill, 2005; Tharenou, 2015), or the overseas pull to weaken their willingness to return home (Jensen and Pedersen, 2007; Baruffaldi and Landoni, 2012).

The issue of influencing factors of overseas students’ return home has been concerned by the academic community, but the existing researches still have its limitations. First of all, most of the theoretical perspectives are based on push-pull theory (Buckner et al., 2022) to analyze the push or pull factors that affect the return of international students. However, the push-pull theory lays too much emphasis on external macro factors and constructs international students as passive selectors driven by external factors, ignoring their subjectivity and initiative when considering whether to return home or not. In addition, the factors influencing the return of overseas students are complex and changeable (Hari et al., 2021). When the political conditions, economic situation, and social environment of the home country and the host country change, the willingness, flow direction, and influencing factors of overseas students will also change (Yıldırım et al., 2021). At present, the impact of the COVID-19 pandemic on the return of overseas students cannot be ignored, but there are few relevant empirical studies (Firang and Mensah, 2022). Although a few existing studies have addressed this topic (Ahmad et al., 2021; Wang, 2021), they are all based on qualitative interviews of small samples, lacking empirical research of large samples. It can be seen that the research on the impact of COVID-19 on the return of overseas students is in the initial stage (Yang et al., 2022).

Because of this, this study believes that it is necessary to seek a new theoretical framework to carry out empirical research on the factors affecting the return of foreign students (Bista et al., 2021). Specifically, the questions that this study wants to explore are: Under the new situation that COVID-19 has not completely dissipated and may continue to affect the international pattern, what choices will overseas students make on whether to return home? What is their intention to return home? What are the factors that affect their return? What factors can predict their return behavior? Furthermore, how will these factors affect the global talents’ flow in the future? What lessons can we learn from it?

In order to answer the above questions, this study took Chinese students studying in the United Kingdom as an empirical case, based on the theory of rational choice, and adopted a mixed research method to explore the return choices of Chinese students and its influencing factors during the special period (2020.03–2020.05) when the COVID-19 was under control in China, while it was spreading dramatically in the United Kingdom. In the context of the global public health crisis, paying attention to this issue will help the government in the post-epidemic era to improve its crisis response and governance capabilities, and provide a realistic basis for relevant departments to improve the management and services for overseas students, help to attract and retain more high-quality overseas talents, and also is of great significance to enhance the country’s international competitiveness. At the same time, this study can also provide reference for other developing countries like China. Finally, it also contributes to the equalization of global talent distribution.

## Theoretical basis and model construction

### Theoretical basis

Rational Choice Theory (RCT) is one of the most important theories in sociology to study individual behavior with economic methods (Bezar et al., 2021). This theory believes that people are purposeful rational actors, and their action decisions in social life are choices made by rational thinking and weighing the advantages and disadvantages. The principle of the actor is to calculate the cost and benefit rationally, to maximize personal interests as much as possible (Massey et al., 1994) and optimize the action utility (Zafirovski, 2003).

As a master of rational choice theory, J. S. Coleman believes that rational behavior involves actors, resources, and interests, as well as social systems and structural constraints that affect actions (Coleman, 1994). Among them, personal interests and structural constraints are the two decisive factors that affect people’s rational choice of actions. Actors are rational people with a purpose. Resources are certain things, including wealth, goods, information, skills, events, etc., which are important conditions for actors to act. Interests constitute the basic motivation of rational actors, which is manifested in certain needs and preferences, including material, spiritual and social needs, and preferences (Coleman, 1994). G. Ritzer believes that individual preferences are not static. External social systems and structures encourage certain actions and inhibit other actions by providing positive and negative rewards and punishment measures (Ritzer and Stepnisky, 2017). To sum up, we can say that rational choice behavior is a purposeful behavior produced by actors under structural constraints, according to their own needs and preferences, using possible resources and weighing the advantages and disadvantages.

Facing the different COVID-19 epidemic situations in China and Britain, international students need rational thinking to decide whether to return to China or not. In other words, they are rational actors. Their interests are expressed as the survival needs under the epidemic situation, that is, the maintenance of life safety and physical health; personal development needs (Mok et al., 2022), such as academic progress, job hunting after graduation, etc., as well as the consideration of the economic input and benefit needed to return to China or not, and the spiritual and emotional needs of family and friendship (Gill, 2005; Jensen and Pedersen, 2007; Baruffaldi and Landoni, 2012; Tharenou, 2015), as well as different national systems (Gribble, 2008; Mohamed and Abdul-Talib, 2020) and social culture (Alberts and Hazen, 2005; Tharenou and Seet, 2014; Bonifazi and Paparusso, 2019). Resources are essential for actors. Without resources, actors lose their ability to act. The resources of overseas students during the COVID-19 epidemic mainly refer to daily necessities, epidemic prevention supplies, economic resources, flight resources, etc. This is an important guarantee for overseas students to continue to survive abroad or return to China. Structural constraints mainly refer to the epidemic control policies of China and the United Kingdom, the management system for overseas students, social norms, and culture, etc. In brief, we believe that under the COVID-19 epidemic situation, whether the overseas students who are rational actors return to China or not are the result of rational calculation. Under the constraints of a certain social structure, for their own interests, according to their own resources, international students comprehensively consider various factors and rationally calculate the costs and benefits of returning to China or not.

In the process of choice, the actors will show different levels of rational pursuit or rational tendency due to their different needs, different resources, and different degrees of structural constraints. According to RCT, the related research, and the pre-investigation of this study, the researchers classify rational tendencies into five types: survival rationality, economic rationality (Diesing, 1950; Lindenberg, 2001), social rationality (Wenjun, 2001; Teimouri et al., 2019), institutional rationality (Redmond, 2004), and development rationality (Lian and Liu, 2019; Ren nad Yu, 2021). Survival rationality pays attention to individual survival and safety first, mainly considering the living conditions of the actors themselves and other actors around them (Coleman et al., 1992). Social rationality emphasizes the realization of the social needs of actors (Hechter and Kanazawa, 1997). Economic rationality aims at maximizing economic benefits (Hechter and Kanazawa, 1997; Nida-Rümelin, 2013). Institutional rationality refers to actors’ judgment on the significance and value of policies, measures, norms, etc., and their consideration of the effect of system implementation, so as to choose which system design to follow (Bigirimana, 2017). Development rationality focuses on considering personal career development prospects and individual growth (Ren nad Yu, 2021).

### Model construction

In order to make the RCT more suitable for this study, the researchers used text analysis and qualitative interviews to extract and purify the influencing factors and dimensions of returning overseas students in the United Kingdom, so as to provide a basis for the preparation of the questionnaire.

#### Network news text collection

Using the Internet search engine, we searched the relevant news in the 2 months from March 2020 to April 2020 with the keywords such as “influencing factors of returning home,” “International students during the COVID-19 pandemic,” and “returning home during the COVID-19 pandemic,” etc., and obtained 80 pages of related news articles in total.

#### Qualitative interview

In this study, semi-structured interviews were conducted with the subjects by means of WeChat chat or telephone, and a total of 12 international students were interviewed (Table 1). The main contents of the interview are: What do you think of the situation of the COVID-19 epidemic prevention and control abroad? Do you want to go back to China? Why do you choose to stay in Britain (or return to China) now? When you decide whether to return to China, what are your main considerations?

**Table 1:** Basic information of interviewed international students.

**No.**	**Gender**	**Major**	**Identity**	**Length of time in the UK**
1	Female	Statistics	Undergraduate	1 year
2	Female	Communication engineering	Master’s degree candidate	half a year
3	Female	Finance	Master’s degree candidate	1 year
4	Female	Sociology	Doctoral student	half a year
5	Male	Nursing	Undergraduate	half a year
6	Female	Clinical medicine	Doctoral student	half a year
7	Male	Control science and Engineering	Doctoral student	2 years
8	Male	Human resource management	Undergraduate	4 years
9	Female	Higher education	Doctoral student	6 years
10	Male	International management	Master’s degree candidate	3 years
11	Male	Marketing	Undergraduate	3 years
12	Female	Social media	Master’s degree candidate	1 year

#### Text data coding and analysis

Based on the RCT, we encode the collected online news and interview texts step by step, abstract and refine the factors and dimensions that affect the return of overseas students in the United Kingdom (Table 2). According to the code, there are 17 factors in five dimensions: survival rationality, economic rationality, social rationality, institutional rationality, and development rationality.

**Table 2:** Coding results of influencing factors of overseas students returning home.

**Dimensions**	**Categories**	**Factors**	**Examples from online news or interviews**
Survival rationality	Life and Risk Factors in Foreign Countries	Protective supplies reserve	Personal protective clothing, face masks, and disinfectant are so hard to buy that no one dares to return to China.
		The supply of daily necessities abroad	The supermarket snapped up badly. Everyone hoarded goods. All the cheap markets in Leicester are closed and supermarket prices are rising. Try to buy more items and store them at a time.
		Epidemic prevention awareness and behavior of foreign people	British people only wear masks when they are sick. Wearing a mask in public places and being abused by passers-by, I usually dare not go out. My neighbor still goes his own way, having a family party on weekends. I am worried that I will be infected by people around me.
		Collective life abroad	I live in a group apartment, where the kitchen and bathroom are shared, which can easily cause cross infection.
		Infection risk when purchasing outside	Every few days, I have to go to Tesco to buy things. There are many people there. The local people do not wear masks. I am very worried.
Social rationality	Social relation factors	Media public opinion	Alarmed by some “discriminatory” comments against overseas students on the Internet, I repeatedly told my parents not to tell others about my return date.
		family relationship, friendship and love	My family advised me to go back as soon as possible. It is safe to return home early. My friend is getting married on May Day and wants me to go back to China to attend his wedding.
Economic rationality	Return cost factors	Transportation cost	Look at the recent air tickets, the lowest starting price is 20,000. Air tickets are expensive and not available. My scheduled flight ticket has to change planes. It is so difficult. Before returning home, you need to isolate yourself for 14 days in advance and report your daily physical condition to the relevant domestic departments before boarding. After returning to China, you cannot go home until 14 days after the quarantine.
		Quarantine expenses	
		time cost	
Institutional rationality	Policy control factors	British epidemic control policy	Britain’s “herd immunization” policy makes people uneasy. Its effect is not optimistic.
		China’s epidemic prevention and control policy	No one can replicate China’s epidemic prevention and control. In this epidemic, China has the lowest mortality rate.
		The policy of the Embassy in the UK to help Chinese students studying abroad	I am so happy that I received a “health package” from my motherland.
		Management policy for international students	My visa has expired, but I can apply for an extension to May before returning to China.
Development rationality	Personal development factors	Academic research progress	The school informed that all courses will be changed to online courses from next month, and I am considering whether to return to China for online courses. If I went back to China, I have to get up in the middle of the night to take online classes. Half of my experiment has been carried out, and the data can only be collected abroad.
		Mentor’s requirements and suggestions	Because I need to hold frequent seminars with my tutor’s team, the teacher suggested that I should not return home for the time being to avoid poor communication.
		Graduation and job hunting	At present, the epidemic situation in foreign countries is not well controlled. I think it is more reassuring to return to China to find a job after graduation. China is developing very well now.

**Figure 1:**
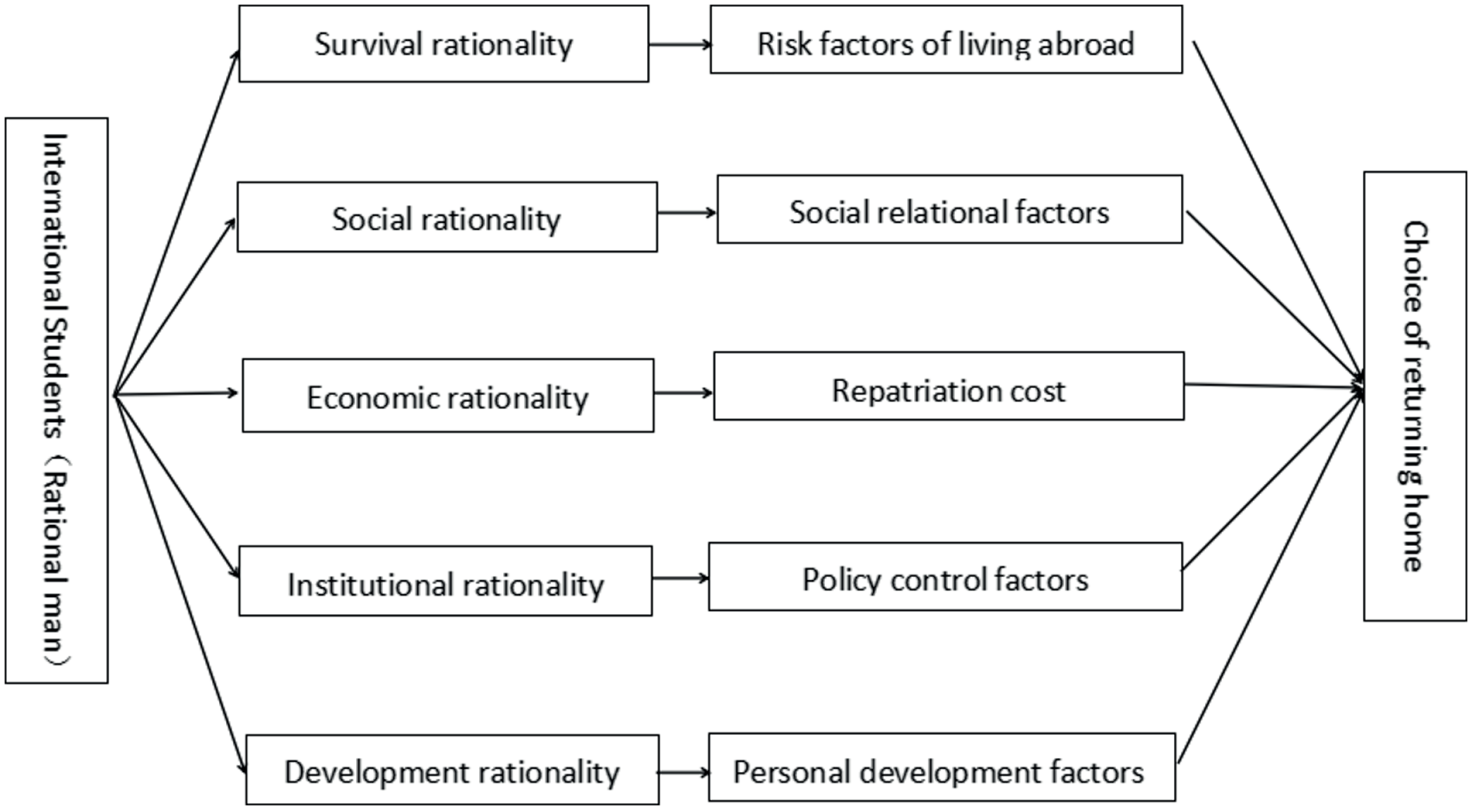
Model of influencing factors of overseas students’ choice of returning home.

In this study, survival rationality factors mainly refer to the living conditions and infection risks of overseas students during the COVID-19 epidemic. Social rationality factors mainly include the social relations, emotional ties, and social public opinion of international students at home and abroad. Economic rationality factors mainly refer to the investment and cost of returning to China, including transportation fees, isolation fees, testing fees, etc. Institutional rationality factors refer to the epidemic prevention and control measures taken by the Chinese and British governments and the management policies for overseas students. Development rationality factors refer to personal academic, career planning, and individual development prospects. During the pandemic, the choice of whether international students return to China is the result of rational calculation. Therefore, it is appropriate to analyze the influencing factors of overseas students’ choice of returning home during the epidemic based on RCT. Combining RCT with qualitative coding results; this study established the following model (Figure 1).

## Materials and methods

### Questionnaire design and test

The research team drafted the first version of the questionnaire according to the qualitative coding results (Table 2), and randomly selected some international students to try it. Taking into account the feedback of international students, the research group further improved and finalized the questionnaire through many discussions. The questionnaire includes three parts: introduction, demographic variables (gender, age, marital status, annual household income, etc.), and the investigation of influencing factors. The research group distributed 300 trial questionnaires and collected 286 questionnaires, of which 252 were valid. The researcher tested the validity and reliability of the questionnaire.

We used SPSS26.0 to conduct principal component analysis (PCA) on 17 items from five dimensions (Table 2) in the questionnaire. Firstly, Bartlett spherical test and KMO value test were performed. The chi-square value of Bartlett’s sphericity test was 1105.769 (sig = 0.000), and the KMO value was 0.746 (>0.7), indicating that the original 17 items were suitable for factor analysis. Secondly, the principal component analysis and the maximum variance method were used to rotate the factor loading matrix. Four factors with eigenvalues greater than 1 were extracted. The cumulative variance contribution rate of the four factors was 65.74%. Items with low scores or unreasonable attribution, such as visa policy, emotional ties of family and friends, and media opinions, were deleted one by one. Principal component analysis was conducted for every item deleted, and finally four dimensions (*Factor1 = survival rationality, Factor2 = institutional rationality, Factor3 = economic rationality, and Factor4 = development rationality*) and 13 items were obtained. The scores of each item are shown in the table.

We calculated Cronbach’s α for this questionnaire, which was 0.885, to test the internal consistency and reliability. Bartlett sphericity test showed *p* < 0.01, KMO was 0.838. The Spearman-Brown split-half reliability coefficient was 0.725, which indicates that the questionnaire has high reliability. The research group ensured the content validity of the questionnaire through network text analysis, qualitative interviews, questionnaire trial, exploratory factor analysis, and discussions among members. In addition, the correlation coefficient between each factor of the questionnaire and the total score is between 0.634 and 0.817, which is higher than the correlation among all factors, reaching a significant level, indicating that the questionnaire has good structural validity. In a word, the reliability and validity of the questionnaire are good, which indicates that the questionnaire is suitable as an instrument for the influencing factors of overseas students returning to China.

### Data

This study takes Chinese international students in the United Kingdom as the research object based on the following two considerations. First, China is the largest source of international students in the United Kingdom. The United Kingdom is the preferred destination country for Chinese students to study abroad. Second, China is one of the largest exporters of international students and the largest developing country in the world. Britain is one of the largest importers of overseas students and an important representative of developed countries. It may be more typical to take Chinese students studying in the United Kingdom as the survey sample. With the help of the Chinese Embassy in the United Kingdom and the Federation of Chinese Overseas Students in the United Kingdom, from the end of April to the beginning of May 2020, we distributed 1,569 online questionnaires through online social media such as WeChat group of overseas students in the United Kingdom and finally collected 1,333 valid questionnaires. The COVID-19 pandemic provided a natural experiment. Conducting research under the pandemic situation can better reflect the true thoughts of the respondents and approach the objective facts. And then, we can also find out what changes will happen to the influencing factors of overseas students returning to China after the outbreak of the COVID-19 pandemic.

In this study, SPSS 26.0 was used for statistical analysis, with the statistical level set at *p* < 0.05 (two-sided). The methods of descriptive statistics and binary Logistic regression analysis are used to reveal the influencing factors of overseas students’ choice of return to China. Stata15.0 performed a robustness test.

Table 3 Descriptive statistics of variables.

**Table 3:** The factor loading matrix.

**The rotated component matrix** ^**a**^				
**Items**	**Components**			
	**Factor1**	**Factor2**	**Factor3**	**Factor4**
Epidemic prevention supplies reserve	0.796			
The supply of daily necessities in the United Kingdom	0.862			
Collective life risks abroad	0.769			
Infection risk when purchasing outside	0.777			
Isolation fee			0.661	
Transportation expenses for returning home			0.794	
Return time cost			0.824	
Academic research progress				0.719
Mentor’s requirements and suggestions.				0.799
Graduation and job hunting				0.521
British epidemic control policies		0.723		
China’s epidemic prevention and control policies		0.837		
The policy of the Embassy in the United Kingdom to help Chinese international students		0.764		

Extraction method: Principal component analysis.

Rotation method: Varimax.

^a^The rotation has converged after six iterations.

### Variable declaration

#### Dependent variable

Return choice is the dependent variable. It refers to whether students studying in the United Kingdom choose to return to China or stay in the United Kingdom when facing different epidemic situations in China and the United Kingdom. This variable is transformed into this question: “Do you choose to return to China? or Have you returned to China?” in the questionnaire. The variable was set as binary dummy variables (choose not to return to China = 0, choose to return to China/have returned to China = 1; Table 4). It needs to be explained that the choice of returning home in this study is not only willingness or tendency but also a factual behavior. In the sample, there are both international students who have returned to China and those who chose to stay in the United Kingdom. This precisely provides us with a situation close to the quasi-natural experiment, which is more conducive to exploring the real factors that affect the return of overseas students.

**Table 4:** Setting and description of related variables.

**Variable type**		**Dimension**	**Variable**	**Variable assignment**
Dependent variable		Choice of returning home	Whether to return to China	Choose not to return to China = 0, choose to return to China = 1
Independent variables	Control variables	Demographic factors	Gender	Male = 0(control group,), female = 1
			Marital status	Single (control group), in love, married
			Annual household income	Less than 100,000 CNY(control group,), 100,000 ~ 200,000 CNY, 200,001 ~ 300,000 CNY, and 300,001 CNY and above
			Length of study abroad	Half a year (control group,) 1 year, 1.5, 2, and 2.5 years, and above
	Main independent variables	Survival rationality (Life and Risk Factors in Foreign Countries)	Insufficient reserve of epidemic prevention supplies	1 (very inconsistent)–5 (very consistent)
			The supply of daily necessities abroad is tight.	1 (very inconsistent)–5 (very consistent)
			Risk of collective life in Britain.	1 (very inconsistent)–5 (very consistent)
			Infection risk when purchasing outside.	1 (very inconsistent)–5 (very consistent)
		Economic rationality (cost factors of returning home)	The transportation cost is high.	1 (very inconsistent)–5 (very consistent)
			The isolation cost is high.	1 (very inconsistent)–5 (very consistent)
			The time cost is high	1 (very inconsistent)–5 (very consistent)
		Institutional rationality (Policy control factors)	The United Kingdom’s epidemic control policy is weak	1 (very inconsistent)–5 (very consistent)
			China’s epidemic prevention and control policies are effective	1 (very inconsistent)–5 (very consistent)
			Embassy in the United Kingdom to help Chinese international students	1 (very unimportant)–5 (very important)
		Development rationality (personal development factors)	Academic research progress	1 (very unimportant)–5 (very important)
			Graduation and job hunting	1 (very unimportant)–5 (very important)
			Mentor’s requirements and suggestions.	1 (very unimportant)–5 (very important)

#### Independent variables

Independent variables are continuous variables, including individual survival factors, cost factors of returning home country, institutional control factors, and personal development factors, which, respectively, correspond to four dimensions: survival rationality, economic rationality, institutional rationality, and development rationality (Table 4). Each variable of each dimension is assigned a value according to Likert scale 1–5, from 1 to 5, which indicates that the influence of this factor on respondents is getting higher and higher. For example, “the transportation expenses borne by returning to China are higher for me,” from 1 (very inconsistent) to 5 (very consistent) means that the higher the degree of conformity with the reality, the stronger the influence of the factors, and the weaker the opposite.

#### Control variables

Control variables are demographic variables, mainly including gender, marital status, length of study abroad, annual household income, etc. (Table 4). Gender is a binary variable, with “male” as the reference variable. Marital status include single, in love, and married, which are classified into three variables, with “single” as the reference variable. Take the annual income of 100,000 as a unit and set it as four classified variables: less than 100,000 CNY, 100,000 ~ 200,000 CNY, 200,001 ~ 300,000CNY, 300,001CNY and above, with “less than 100,000 CNY” as the reference variable. The duration of studying abroad is 6 months as a unit, and it is set into five categories: half a year, 1 year, 1.5, 2, and 2.5 years, with “half a year” as the reference variable. It should be noted that this study does not discuss demographic control variables.

## Results and discussion

### Preliminary analyses

Table 5 presented the differences of the observed variables in gender, Marital status, household income, Length of study abroad, Academic identity, and Age. Results of independent-sample *t*-test indicated that there were significant gender differences in the survival rationality and economic rationality. There were no significant differences in the scores across the four dimensions, whether the students were single or not.

**Table 5:** Descriptive statistics and mean differences.

Variables	Items	Survival rationality (M±SD)	Economic rationality(M±SD)	Institutional rationality (M±SD)	Development rationality(M±SD)
Gender	Male (*n* = 398)	2.915 ± 1.100	3.648 ± 0.930	3.498 ± 1.039	3.048 ± 0.966
	Female (*n* = 935)	3.090 ± 1.022	3.795 ± 0.881	3.575 ± 0.948	3.150 ± 0.922
	*t*	−2.726^**^	−2.731^**^	−1.266	−1.828
Marital status	Single (*n* = 838)	3.062 ± 1.045	3.766 ± 0.895	3.566 ± 0.965	3.125 ± 0.937
	In love or married (*n* = 495)	2.998 ± 1.056	3.726 ± 0.904	3.529 ± 0.996	3.110 ± 0.935
	*t*	1.081	0.781	0.656	0.293
					
Annual household income	Less than 100,000 CNY (*n* = 234)	3.117 ± 1.105	3.929 ± 0.867	3.560 ± 1.019	3.083 ± 1.013
	100,000~200,000 CNY (*n* = 403)	3.054 ± 1.029	3.783 ± 0.875	3.591 ± 0.948	3.203 ± 0.905
	200,001~300,000CNY (*n* = 289)	3.130 ± 0.992	3.694 ± 0.905	3.586 ± 0.986	3.150 ± 0.939
	300,001CNY and above (*n* = 407)	2.910 ± 1.067	3.657 ± 0.919	3.485 ± 0.972	3.036 ± 0.914
	*F*	3.256^*^	5.153^**^	0.982	2.389
Length of study abroad	Half a year (*n* = 340)	3.139 ± 1.030	3.756 ± 0.914	3.571 ± 0.977	3.115 ± 0.968
	A year (*n* = 562)	3.074 ± 1.027	3.724 ± 0.873	3.610 ± 0.939	3.174 ± 0.886
	One and a half years (*n* = 95)	2.971 ± 1.033	3.7860 ± 0.928	3.393 ± 1.026	3.074 ± 1.027
	Two years (*n* = 84)	2.961 ± 1.136	3.861 ± 0.881	3.464 ± 0.944	3.008 ± 1.039
	Two years and above (*n* = 252)	2.872 ± 1.0831	3.755 ± 0.928	3.487 ± 1.042	3.060 ± 0.930
	*F*	2.753^*^	0.486	1.616	1.088
Academic identity	Undergraduate (*n* = 409)	3.143 ± 1.032	3.599 ± 0.970	3.468 ± 0.916	2.861 ± 0.972
	Master's degree candidate (*n* = 758)	3.132 ± 1.007	3.810 ± 0.838	3.604 ± 0.849	3.297 ± 0.860
	Doctoral student (*n* = 166)	2.400 ± 1.296	3.965 ± 0.856	3.342 ± 0.954	3.080 ± 0.914
	*F*	1.057	4.151^***^	3.585^***^	11.023^***^
					
Age	0-18 years old (*n* = 20)	2.963 ± 1.110	3.600 ± 0.714	3.083 ± 0.990	2.683 ± 1.100
	19-25 years old (*n* = 1069)	3.053 ± 1.053	3.718 ± 0.907	3.575 ± 0.973	3.114 ± 0.928
	26-35 years old (*n* = 208)	2.968 ± 1.026	3.941 ± 0.834	3.463 ± 1.009	3.240 ± 0.937
	36-40 years old (*n* = 19)	3.171 ± 1.024	3.719 ± 1.026	3.632 ± 0.830	2.702 ± 0.942
	Over 40 years old (*n* = 17)	2.898 ± 1.072	3.706 ± 0.904	3.686 ± 0.786	2.941 ± 1.042
	*F*	0.467	2.853^*^	1.843	3.078^*^

^***^p < 0.001, ^**^p < 0.01, and ^*^p < 0.05.

Furthermore, the results of one-way ANOVA indicated that survival rationality and economic rationality showed significant income level differences. However, institutional rationality, and development rationality showed no significant income level differences. The results also showed that there was no significant difference among economic rationality, institutional rationality, and developmental rationality in the Length of study abroad. Whereas only survival rationality showed a significant Length of study abroad difference. Except survival rationality, economic rationality, institutional rationality, and development rationality all showed Degree-Level differences. In addition, survival rationality and institutional rationality showed no significant age differences. Whereas economic rationality and development rationality showed a significant age differences.

### Normal distribution and collinearity test

To ensure the validity of the estimate, we conducted normality and multicollinearity test. Histogram, P–P diagram, Q-Q diagram, skewness, and kurtosis were used to test the normality of 13 variables and four dimensions: survival rationality, economic rationality, institutional rationality, and development rationality. The absolute values of the skewness coefficient and kurtosis coefficient are less than 1.96 (Joanes and Gill, 1998).The test results show that the data conform to normal distribution. Tolerance and variance inflation factor (VIF) are used to test the multicollinearity amid independent variables. The tolerance is greater than 0.1 and the VIF values are all less than 3 (Midi et al., 2010). This shows that there is no multicollinearity problem, and the data could be analyzed by Binary Logistic regression.

### Binary logistic regression analysis of influencing factors of international students’ choice of returning to China

The Hosmer-Lemeshow test was conducted to ensure the goodness of fit of the model. The result shows that *p* = 0.452 (>0.05), which indicates that the overall GOF of the model is good and effective. Table 6 shows the binary logistic regression results of the impact of various factors on the return choice of foreign students. According to the results of regression analysis, only gender variables have a significant impact on the return choice of foreign students among the demographic variables. Specifically, females are more likely to choose to return to China than males. Among the main independent variables, the regression coefficients of six variables, such as epidemic prevention supplies reserves, supplies of daily necessities, transportation costs, isolation fees, anti-epidemic policies in the United Kingdom, and graduation and job hunting demand, are significant, indicating that the above variables have a strong predictive power on the return behavior of Chinese international students. The OR value in the table is the ratio that the dependent variable increases by one or more levels every time the independent variable changes by one unit (Midi et al., 2010).

**Table 6:** Binary logistic regression analysis of influencing factors of overseas students’ choice of returning to China.

**Dimensions**	**variables**		**B estimated value**	**Wald value**	**Sig**	**OR value**
Demography	Gender	Male	0	—	—	1
		Female	0.688	5.382	0.020^*^	1.990
	Marital status	Single	0	—	—	1
		In love	0.150	0.337	0.561	1.162
		Married	0.059	0.010	0.920	1.061
	Annual household income	Less than 100,000 CNY	0	—	—	1
		100,000 ~ 200,000 CNY	−0.378	0.809	0.368	0.685
		200,001 ~ 300,000CNY	0.012	0.001	0.978	1.012
		300,001CNY and above	0.543	1.881	0.170	1.721
	Length of study abroad	half a year	0	—	—	1
		a year	−0.421	1.968	0.161	0.656
		One and a half years	−1.152	3.360	0.067	0.316
		2 years	−0.224	0.166	0.684	0.800
		2 years and above	0.006	0.000	0.987	1.006
Survival rationality	Life and Risk Factors in Foreign Countries	Protective supplies reserve	−0.339	4.560	0.033^*^	0.712
		Daily necessities supply	0.402	7.302	0.007^**^	1.495
		Collective life risk	0.033	0.064	0.800	1.034
		Outgoing purchasing risk	0.191	1.594	0.207	1.210
Institutional rationality	Policy control factors	Foreign prevention and control policies	0.521	12.801	0.000^***^	1.684
		China’s prevention and control policies	0.071	0.236	0.627	1.074
		Embassy assistance	−0.155	1.223	0.269	0.856
Economic rationality	Cost factors of returning home	Transportation expenses	−0.481	16.685	0.000^***^	0.618
		Return time cost	−0.170	1.609	0.205	0.844
		Isolation fee	−0.354	7.613	0.006^**^	0.702
Development rationality	Personal development factors	Academic research progress	−0.065	0.264	0.607	0.937
		Graduation and job hunting	0.508	18.394	0.000^***^	1.661
		Mentor’s requirements and suggestions	−0.049	0.271	0.602	0.953
Constant			−3.410	16.684	0.000	0.033

The first classification independent variable in demographic variables is the reference group, so no value is displayed in the table. ^***^p < 0.001, ^**^p < 0.01, and ^*^p < 0.05.

First of all, in terms of individual survival factors, that is, in the consideration of survival rationality, the storage of epidemic prevention supplies and the supply of daily necessities can predict the return behavior. The less protective supplies overseas students have, the less likely they are to return to China. The recognition degree of insufficient reserve of protective equipment increases by one unit, and the possibility of returning to China decreases by 28.8% (OR = 0.712). The study showed that the main transmission routes of novel coronavirus include droplets, aerosol, and contact transmission (Zhang et al., 2020). In other words, novel coronavirus may be infected by coughing, sneezing, and rubbing eyes with hands that have been exposed to the virus. Therefore, masks, goggles, protective clothing, disinfectant, alcohol, and gloves are the “necessities” to effectively block novel coronavirus (Pan et al., 2022). In addition, the road to return to China is long and it takes a long time to be trapped with strangers in a closed cabin. On the way, international students have to make multiple transfers and pass through the crowded airports. Protective articles such as masks are essential. However, at the early stage of the outbreak of COVID-19 in the United Kingdom, there was a lack of protective equipment and medical resources. This made students studying in the United Kingdom not qualified for long-distance travel back to China, which hindered their return.

The supply of daily necessities can promote the return behavior of overseas students in Britain, and the OR value is 1.495. For every unit that the assessment of the shortage of daily necessities rises, the possibility of overseas students returning to China increases by 49.5%. Daily necessities, such as grain, oil, meat, milk, and eggs, are the basic material guarantee to maintain people’s survival and life health. Affected by the spread of the epidemic and control measures, Britain’s material production is weak, logistics is not smooth, and the supply of daily necessities is tight (Cai and Luo, 2020). Moreover, going out to purchase will also face a great risk of aggregation infection, making the supply of daily necessities even worse (Balmford et al., 2020). It is reported that in the early stage of the outbreak of the COVID-19 epidemic, British people went to supermarkets without masks to rush for supplies, and many supermarkets were snapped up. In contrast, China’s epidemic control has achieved good results, and socioeconomic development has been able to operate normally. The supply of materials is sufficient, and people’s life is stable and orderly. Naturally, students studying in Britain are more inclined to return to China.

On the level of individual investment and cost, that is, the consideration of economic rationality, the transportation fee, and the isolation fee have a significant negative impact on returning to China for students in Britain. High transportation and isolation fees will significantly reduce the willingness and behavior of overseas students to return home. For each additional unit of transportation cost, the possibility of returning to China will decrease by 38.2% (OR = 0.618). The possibility of returning home will be reduced by 29.8% (OR = 0.702) for each additional unit of overseas students’ consideration of returning home isolation costs. According to RCT, the number of resources that actors master affects the achievement of their goals. People with more resources are easier to achieve their goals, while people with fewer resources are difficult to achieve their goals. Economic capital and flight resources play an important role in the process of returning to China, which are important guarantees to facilitate the return of overseas students. Affected by the COVID-19 epidemic, the resources for return flights are tight. The demand for flight resources exceeds the supply. The cost of returning to China has soared. For example, during the outbreak of the epidemic in March–May 2020, the price of air tickets back to China from all over Europe rose by 173.5%. Recently, Turkish Airlines’ flight ticket to China exceeded 250,000 yuan, once again refreshing the “ceiling” of international ticket prices. Even so, it is often hard to get a return ticket. In addition, due to the epidemic, there is a lack of direct flights between China and Britain. Transfer and isolation will also increase the cost of returning home. These are important factors that hinder students from returning to China. In the sample of this study, the proportion of students who have returned to China is very low, only 91. Most of them (*N* = 1,242) chose to stay in the United Kingdom. The cost has become the biggest constraint on whether overseas students return home or not.

Thirdly, in the aspect of institutional control, that is, the consideration of institutional rationality, the variable of epidemic prevention and control policies in the United Kingdom has a significant predictive effect on overseas students’ return behavior. As shown in Table 6, the epidemic control policies in Britain can promote Chinese students to return home. For every unit of dissatisfaction with the British epidemic prevention policies, the possibility of overseas students returning to China increased by 68.4% (OR = 1.684). Britain’s epidemic prevention and control policies have the greatest influence on overseas students returning home. RCT holds that institutional arrangements or social norms can encourage or weaken the behavior of actors (Ritzer and Stepnisky, 2017). The British government initially tried to end the epidemic through the policy of “herd immunity,” which led to a surge in the number of confirmed cases (Hu et al., 2022). The implementation of the policy in the United Kingdom and the severe epidemic situation caused by it are bound to cause resentment and panic among international students, which is an important thrust for international students to choose to return. Although the influence of China’s prevention and control policy in this study is not significant, we also learned in the interview that better epidemic control in China was the main reason why respondents chose to return. Relevant surveys also show that the good effect of epidemic prevention and control in China is the first reason why overseas students choose to return to China.

Finally, in terms of personal development and career planning, that is, the consideration of development rationality, the demand for job hunting after graduation is an important factor to promote overseas students to return. Every time the importance of job-seeking needs in the decision-making process of overseas students’ return to China increases by one unit, the possibility of returning home increases by 66.1% (or = 1.661). Affected by the COVID-19 epidemic, the international economic situation has generally declined. More and more Chinese overseas students choose to return to China for job hunting and development after graduation. According to the survey, after the outbreak of the epidemic, the number of overseas students returning to China to apply for jobs in 2020 increased by 67.3% year on year. Studies have shown that the first consideration for international students to return home is their personal development prospects. International students generally invest lots of time and economic costs during their study abroad, so they attach great importance to opportunities and platforms for personal development. However, a safe and stable environment is the premise and basic guarantee for personal development. During the epidemic period, the policy solutions that governments enacted were different, which brought different social living environment and economic development. Therefore, the employment opportunities and development prospects of overseas students in different countries will be different. The epidemic situation in China has been basically controlled, social life is stable, and economic development is improving. Both the security of the living environment and good development opportunities has a strong attraction for international students.

The above is the analysis of each significant predictive variable one by one. In order to investigate which rational choice is more influential in the choice of returning home, we calculated the total average of each variable and then processed them by binary Logistic regression (Table 7).

**Table 7:** Binary logistic regression results of the impact of various dimensions on the return choice of overseas students in the United Kingdom.

	Variables	B estimated value	Wald value	Sig	OR
Core independent variable	Survival rationality	0.324	6.503	0.011^*^	1.383
	Economic rationality	−1.053	61.328	0.000^***^	0.349
	Institutional rationality	0.365	6.311	0.012^*^	1.440
	Development rationality	0.317	5.169	0.023^*^	1.373
Control variable	Male	0	—	—	1
	Female	0.575	4.094	0.043^*^	1.777
	Single	0	—	—	1
	In love	0.071	0.081	0.776	1.074
	Married	0.047	0.007	0.934	1.048
	Less than 100,000 CNY	0	—	—	1
	100,000 ~ 200,000 CNY	−0.355	0.745	0.388	0.701
	200,001 ~ 300,000CNY	0.047	0.013	0.908	1.048
	300,001CNY and above	0.703	3.491	0.062	2.019
	half a year	0	—	—	1
	A year	−0.438	2.265	0.132	0.645
	One and a half years	−0.896	2.412	0.120	0.408
	2 years	−0.269	0.247	0.619	0.764
	2 years and above	−0.051	0.023	0.881	0.950
Constant	−2.730	14.139	0.000	0.065	

The first classification independent variable in demographic variables is the reference group, so no value is displayed in the table. ^***^p < 0.001 and ^*^p < 0.05.

Binary Logistic regression shows that the four dimensions can significantly predict the overseas students’ return behavior. Institutional rationality, survival rationality, and development rationality play an important role in promoting the return of overseas students, while economic rationality works in the opposite direction. Among them, institutional rationality has the greatest influence. For each additional unit in the consideration of system and policy, the possibility of overseas students returning to China will increase by 44%. Survival rationality is also an important driving force for international students to choose to return. The possibility of overseas students returning to China will increase by 38.3% for each additional unit in consideration of survival factors. The influence of personal development on the choice of returning home cannot be ignored. For each additional unit of personal development, the possibility of overseas students returning to China increases by 37.3%. The more personal development is considered, the greater the possibility of overseas students returning to China. Economic rationality is the biggest obstacle for international students to return to China. For each additional unit of consideration of economic rationality, the possibility of international students returning to China will decrease by 65.1%. The more international students consider the cost, the less likely they are to return to China.

### Robustness test

We used Stata15.0 to conduct *B-P* test and *White* test to see whether the data had heteroscedastic problems. The results showed that the data had heteroscedasticity [chi2(1) = 87.45, *p* = 0.000; chi2(14) = 102.19, *p* = 0.000]. In order to eliminate heteroscedastic interference and test the robustness of the results (Greene and Hensher, 2007), statistical inference based on heteroscedastic robust standard errors (Koenker and Bassett, 1982) is chosen in this paper (Table 8). Comparison shows that the sign direction and significance of explanatory variables remain unchanged. The values vary slightly within a very small range. This shows that the original model is set correctly and the regression results are robust and reliable.

**Table 8:** Robustness test of related variables.

**Variables**		**Coef**	**Ordinary standard error (Robust standard error)**	
Dependent variable	Choice of returning home		*z*	*p*
Control variables	Gender	0.668	2.290(2.230)	0.022(0.026)
	Marital status	0.122	0.500(0.409)	0.619(0.624)
	Annual household income	0.271	2.200(2.200)	0.068(0.069)
	Study abroad duration	0.038	0.430(0.410)	0.667(0.679)
Independent variables	Protective supplies reserve	−0.314	−1.980(−1.830)	0.047(0.068)
	Daily necessities supply	0.397	2.680(2.620)	0.007(0.009)
	Collective life risk	0.028	0.220(0.230)	0.8250.822)
	Outgoing purchasing risk	0.167	1.130(1.310)	0.259(0.191)
	Foreign prevention and control policies	0.511	3.580(3.370)	0.000(0.001)
	China's prevention and control policies	0.079	0.550(0.570)	0.581(0.566)
	Embassy assistance	−0.146	−1.050(−1.180)	0.292(0.237)
	Transportation expenses	−0.492	−4.250(−4.890)	0.000(0.000)
	Return time cost	−0.153	−1.160(−1.200)	0.245(0.229)
	Isolation fee	−0.346	−2.690(−2.510)	0.007(0.012)
	Academic research progress	−0.084	−0.670(−0.730)	0.506(0.464)
	Graduation and job hunting	0.501	4.220(4.030)	0.000(0.000)
	Mentor's requirements and suggestions	−0.051	−0.560(−0.620)	0.576(0.535)
	_cons	−5.109	−5.030(−4.900)	0.000(0.000)
Control variables	Gender	0.556	1.990(1.950)	0.047(0.049)
	Marital status	0.077	0.320(0.310)	0.747(0.754)
	Annual household income	0.342	2.890(2.690)	0.066(0.067)
	Study abroad duration	0.029	0.340(0.320)	0.733(0.748)
Dimensions	Survival rationality	0.328	2.590(2.620)	0.01(0.009)
	Economic rationality	−1.036	−7.830(−8.620)	0.000(0.000)
	Institutional rationality	0.374	2.580(2.620)	0.01(0.009)
	Development rationality	0.284	2.080(2.140)	0.037(0.033)
	_cons	−4.461	−4.840(−4.830)	0.000(0.000)

In brackets are the values after the robustness test.

## Conclusions and policy implications

### Conclusion

Based on the RCT, this study adopts the mixed research method to explore the influence degree and effect of different factors on overseas students’ return behavior. The main findings are as follows.

The results of the qualitative interview showed that at the beginning of the outbreak of the COVID-19 pandemic in the United Kingdom, affected by the risks of daily life and the epidemic control policies in the United Kingdom, international students are more inclined to flee Britain and return to China. However, due to the high economic cost and the lack of flight resources, most international students are powerless to return to China. This is also confirmed by the descriptive statistical results of the samples. In the sample, only 91 international students were able to return to China at that time, and the rest had to choose to stay in the United Kingdom. But this does not mean that they have no intention to return to China, but that they do not have the conditions and resources to do so. Therefore, remaining in the United Kingdom is the result of a rational choice made by most overseas students with limited resources and conditions when facing the epidemic. Although this is a helpless choice, it may also be the best choice at that time.

The binary logistic regression results show that in the secondary indicators, six variables, such as the reserve of protective equipment, the supply of daily necessities, the transportation cost, the isolation expenses, the epidemic prevention policies in the United Kingdom, and the demand for graduation and job hunting, have a significant impact on the return choice of overseas students in the United Kingdom. Britain’s epidemic prevention policy is the biggest thrust for overseas students to return to China. The transportation cost is the biggest resistance for overseas students to return to China. This also confirmed the information we got in the interview.

From the perspective of dimension, institutional rationality, survival rationality, development rationality, and economic rationality can significantly predict the overseas students’ return behavior. Institutional rationality is the biggest thrust for overseas students in Britain to return. Dissatisfaction with Britain’s anti-epidemic policy and its effect makes international students want to return to China, where the Chinese government has been actively and strictly fighting the COVID-19. Economic rationality is the biggest obstacle for overseas students to return to China. The high cost of returning and the shortage of flight resources hindered the return behavior of international students. Survival rationality is the fundamental driving force for overseas students to choose to return. The survival risks and living difficulties in the UK urge overseas students to return to China where their living environment is safer. Development rationality is also an important driving factor for international students to choose to return to China. Considering the epidemic prevention and control policies and situations in foreign countries, more international students choose to go back to China for job hunting and development. In this regard, we can also find that the concerns of overseas students have changed from the primary consideration of personal development prospects to more attention to national epidemic control policies and their effects under the global public health crisis, reflecting the change from development rationality to institutional rationality. With the improvement of China’s social and economic development level, in the case of relatively small differences in personal development prospects, international students prefer to choose China with better epidemic control and a safe and stable social living environment to develop.

In short, human rationality is not one-dimensional, but multi-dimensional. Whether overseas students choose to return to China is not only affected by survival factors but also restricted by institutional arrangements such as epidemic control policies, as well as the individual’s development prospects at home and abroad and whether the cost of migration is higher than that of those who stay overseas.

### Policy implications

Through empirical analysis, we have made clear the main influencing factors of overseas students’ choice of returning home during the major public health crisis. Accordingly, we can provide some reference suggestions for the government or relevant departments on how to attract talents back to China and improve the service for overseas students, so as to enhance the country’s talent advantage and strengthen its strategic position in the future international competition. In this regard, we provide the following suggestions for the Chinese government or relevant departments. Although the proposal is aimed at China, other similar developing countries can learn from it to promote the balance of global talent distribution.

#### The government should continue to implement the policy of appropriate prevention and control of the epidemic

In this study, institutional rationality is the biggest thrust for overseas students to return. Among them, the epidemic prevention policies of China and Britain are the main consideration factors for overseas students to return. Britain’s initial anti-epidemic policy was relatively passive, which increased the insecurity of Chinese students and adversely affected their daily life. By contrast, living in China is safer, and international students are more inclined to return to China. In the interview, some interviewees also said that the good effect of epidemic control in China was the main reason why they chose to return. Therefore, the prevention and control of the COVID-19 epidemic is the fundamental guarantee to attract talents to return. The government should adhere to the general policy of “dynamic clearance” and continue to implement the anti-epidemic policies of strict prevention and control. This is a fundamental measure to reduce the risk of people’s survival and life, effectively ensure the safety and health of the public, and a strong guarantee for the sound and orderly development of social economy.

#### The government should seek a dynamic balance between epidemic prevention and socioeconomic development

Effective epidemic prevention and control measures can provide a relatively safe and stable environment for people’s normal life and socioeconomic development, but related research also shows that some restrictive epidemic prevention and control measures are negatively correlated with the economic growth rate of cities (Shi et al., 2022). Therefore, the government should take comprehensive consideration and make a top-level design to efficiently coordinate epidemic prevention and control with socioeconomic development. In other words, on the premise of ensuring people’s life safety and daily life, the government should strengthen the social and economic support strategy and combine epidemic restriction, health protection measures, and social and economic support policies^1^ to seek a dynamic balance between epidemic prevention and socioeconomic development. In this way, the safe living environment and stable economic development are more attractive for overseas students to return to serve in China.

#### The government and relevant departments should actively optimize the development environment for returned overseas students

This study found that job hunting after graduation is an important factor that cannot be ignored to promote overseas students to return. Therefore, while doing a good job in epidemic prevention and control and creating a safe and stable social living environment, the government and other relevant departments should also optimize the development environment for overseas students returning to China and actively respond to the “returning tide” of overseas students in the post-epidemic era, so as to not only attract talents but also retain them. For example, the government and other relevant departments should improve and publicize the policy of overseas students returning to China for employment and entrepreneurship, and implement classified and hierarchical management of overseas students. To provide more job information and create more job opportunities for overseas students who have returned to China to apply for jobs. International students are encouraged to start their own businesses and given preferential subsidies in terms of entrepreneurship and innovation, such as providing entrepreneurship funds or issuing entrepreneurial risk subsidies. The government should actively build a high-quality platform for international students committed to scientific research. In addition, in the construction of cultural environment, the government should make efforts to create a more humane environment for career development and improve the acculturation ability of overseas students after returning to China.

#### The government or relevant departments should provide targeted assistance to overseas students and improve the quality of services for overseas students

The government or relevant service departments should pay more attention to overseas students and provide assistance or subsidies within the scope of the government’s ability. For example, the relevant departments can help international students buy daily necessities through donations, fund-raising, or issuing consumption vouchers, to ensure their basic life. It is also important to regularly monitor the mental health condition of the students. We suggest the government provide mental health support services and open psychology clinics for international students. These services should be provided on both an informal and formal basis as well because those students who are unable to visit the clinic physically can get mental support on call or chat online (Irfan et al., 2021). In terms of the return cost, on the one hand, the government should strictly rectify the high-priced air ticket market, crack down on touts high-priced scalping and speculation, and reduce the air ticket price; on the other hand, the government can also provide phased and bulk green channels for Chinese students to return to China through charter flights or government procurement of services. For repatriation, isolation costs, subsidies, deductions, or other forms of labor transfer are used. For example, after the quarantine, international students can obtain a reduction of isolation fees by completing a certain amount of volunteer services.

### Limitations

We are aware that our research may have two limitations. First, this study utilized convenience sampling to collect data online from several WeChat groups for overseas students in the United Kingdom, and the external validity of the sample remains to be verified. Therefore, caution should be used when generalizing the findings of this study to other groups. To address this limitation, future research should be undertaken to use random or stratified sampling. It is also suggested to carry out transnational or cross-cultural research in the future to examine the model in diverse groups, especially in other countries or in different cultures.

Second, our data and results somewhat lacking in timeliness to some extent. The study was conducted from March to May 2020 when the COVID-19 was under control in China, while it was spreading dramatically in the United Kingdom. Now, although the epidemic is still going on, it is different from the international situation 2 years ago. The COVID-19 pandemic is dynamic. The control policies vary among different countries. The pattern of international socio-economic development is also changing. The factors influencing the return of overseas students are complex and changeable. We suggest future research should focus on the changing process of influencing factors of returning home as the epidemic develops. For example, conducting follow-up studies or collecting data throughout the year, which may produce better and more generalizable results.

## Data availability statement

The raw data supporting the conclusions of this article will be made available by the authors, without undue reservation.

## Ethics statement

The studies involving human participants were reviewed and approved by Ethics Committee for Scientific Research of Nanjing Normal University. The patients/participants provided their written informed consent to participate in this study.

## Author contributions

KMZ and NZ were responsible for the overall development of this study, including the planning of sample collection, data analysis, writing, and polishing of the manuscript. KSZ was in charge of the data collection, the construction of the research framework, and analysis of this study. All authors contributed to the article and approved the submitted version.

## Funding

This research was funded by CSC—China Scholarship Council (Grant No. 20200686022).

## Conflict of interest

The authors declare that the research was conducted in the absence of any commercial or financial relationships that could be construed as a potential conflict of interest.

## Publisher’s note

All claims expressed in this article are solely those of the authors and do not necessarily represent those of their affiliated organizations, or those of the publisher, the editors and the reviewers. Any product that may be evaluated in this article, or claim that may be made by its manufacturer, is not guaranteed or endorsed by the publisher.
